# A review of angle kappa and multifocal intraocular lenses and their effect on visual outcomes

**DOI:** 10.1111/aos.17561

**Published:** 2025-08-30

**Authors:** Thomas Kohnen, Viswanathan Ramasubramanian, Rajaraman Suryakumar

**Affiliations:** ^1^ Department of Ophthalmology Goethe University Frankfurt Germany; ^2^ Alcon Research LLC Fort Worth Texas USA

**Keywords:** angle kappa, cataract surgery, intraocular lens design, optical quality, visual disturbances

## Abstract

Although most patients are satisfied with their vision after multifocal intraocular lens (IOL) implantation, dissatisfaction has been reported for various reasons, including poor visual outcomes and visual disturbances. Many published reports hypothesise that preoperative angle kappa may be an associated factor in patient dissatisfaction. Therefore, there is considerable interest in understanding the connection between preoperative angle kappa and visual outcome after multifocal IOL implantation. This narrative review presents clinical data on angle kappa and its relationship to visual outcomes, visual quality, IOL position and visual disturbances after cataract surgery and multifocal IOL implantation. Although recent advances improved objective measurement of angle kappa, inconsistencies in reporting angle kappa make comparison across studies and patient populations difficult. The consensus among multiple studies was that angle kappa did not influence visual and refractive outcomes following multifocal IOL surgery. However, there were conflicting reports on the correlation between angle kappa and subjective visual quality and patient‐reported visual disturbances. Differences in study design, patient characteristics and multifocal IOL characteristics may explain these discrepancies. IOL orientation, tilt and decentration may be affected by angle kappa and likely contribute to some visual disturbances. Additional modelling studies using multifocal IOLs and ocular biometry could improve our understanding of the relationship between angle kappa and IOL alignment. Accurate assessment of preoperative angle kappa in patients with cataracts is important for successful outcomes and patient satisfaction.

## 1 INTRODUCTION

The first concept of multifocal intraocular lenses (MIOLs) originated in the 1980s (Hoffer & Savini, 2014). Since then, significant improvements in both optical design and lens materials have been achieved (Zvornicanin & Zvornicanin, 2018). The underlying optical design of MIOLs is refractive or diffractive and attempts to provide simultaneous vision at multiple distances (Alio et al., 2017; Zvornicanin & Zvornicanin, 2018), a visual benefit that likely minimises the need for additional vision correction for near and intermediate viewing distances (Cillino et al., 2008; Modi et al., 2021; Zhao et al., 2010). Simultaneous vision may be achieved by sophisticated optical designs that redistribute light energy at multiple focal points, creating a multifocal effect (Alio et al., 2017; Zvornicanin & Zvornicanin, 2018). Although most patients are satisfied with visual outcomes after MIOL implantation (Cillino et al., 2008; Modi et al., 2021), some report dissatisfaction. Complaints include, but are not limited to, reduced contrast (Calladine et al., 2012; Cillino et al., 2008); blurred vision (de Vries et al., 2011; Woodward et al., 2009) and visual disturbances, such as glare, halos and starbursts (Calladine et al., 2012; Cillino et al., 2008; De Rojas et al., 2023; Moshirfar et al., 2023; Woodward et al., 2009). Residual refractive error reported by clinicians may contribute to patient dissatisfaction (Woodward et al., 2009). The cause of this dissatisfaction is thought to be multifactorial (de Vries et al., 2011); however, many published reports hypothesise that preoperative angle kappa may be an associated factor (Karhanova et al., 2013; Karhanová et al., 2015; Prakash et al., 2011; Tchah et al., 2017). The purpose of this narrative review is to present an overview of the clinical literature on angle kappa and its relationship to visual outcomes, optical quality, IOL position and visual disturbances after multifocal cataract surgery. In addition, this review will highlight clinical relevance for patients with cataracts who will receive multifocal IOLs.

## 2 PART I. ANGLE KAPPA: MEASUREMENT, DISTRIBUTION AND ASSOCIATED FACTORS

**FIGURE 1:**
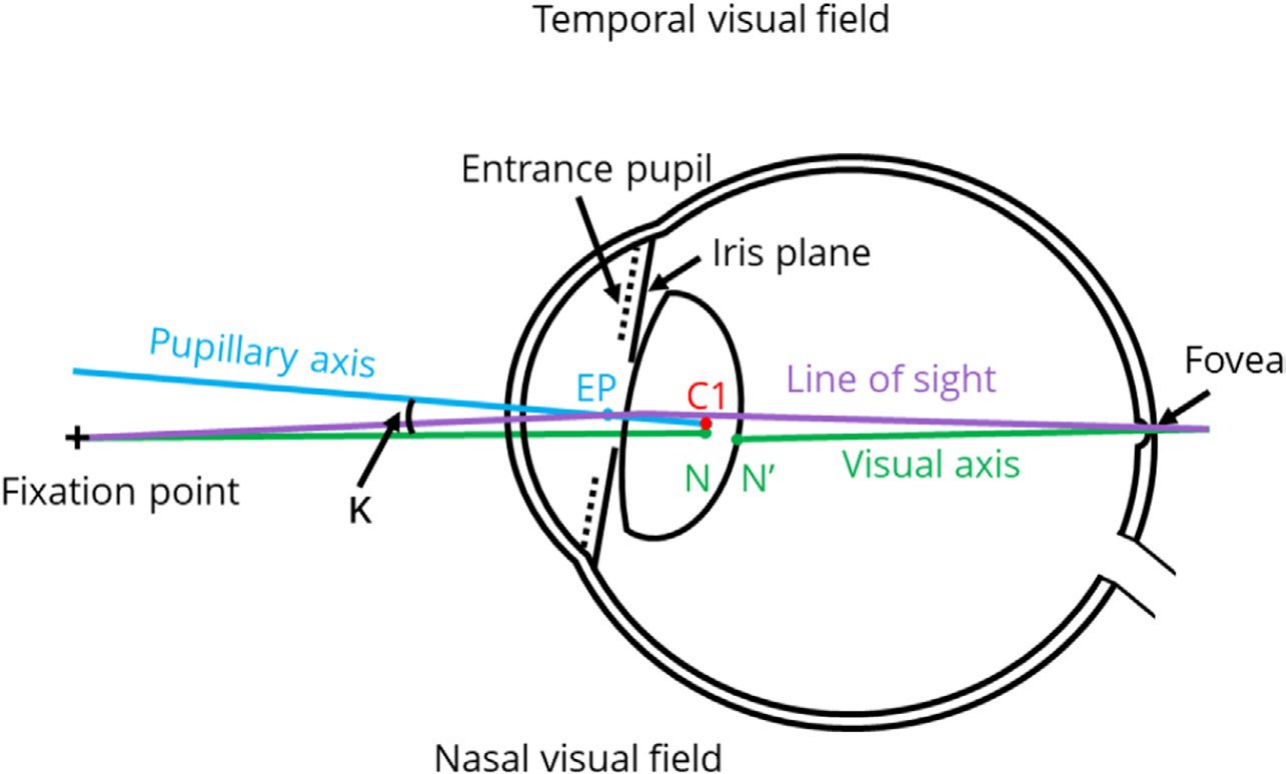
Axes and angles of the human eye. C1, corneal centre of curvature; EP, entrance pupil; K, angle kappa; N and N′, the first and second nodal points.

Several attempts have been made to describe the optical properties of the human eye using optical models (Atchison & Smith, 2000; Bennett & Rabbetts, 2007). These models were constructed at various levels of sophistication by incorporating surface curvatures, axial distances, refractive indices, apertures, axes and angles of various anatomical structures of the eye (Chang & Waring, 2014). Because the eye is not a centred optical system (Atchison & Smith, 2000; Chang & Waring, 2014; Le Grand & El Hage, 1980), the optical axis cannot accurately describe the eye's optics; therefore, alternative axes were described. The pupillary axis is the line perpendicular to the anterior cornea passing through the centre of the entrance pupil and the centre of curvature of the cornea (Atchison & Smith, 2000). The entrance pupil is the image of the iris and is 13% larger due to the magnifying effects of the cornea (Aguirre, 2019). The line joining the fixation point to the fovea passing through the primary and secondary nodal points (N and N′) of the eye is the visual axis. The angle between the pupillary and visual axes is angle kappa (Figure 1). Because of the temporal position of the fovea relative to the pupillary axis, the corneal light reflex is slightly nasal to the centre of the cornea. This is called positive angle kappa, a commonly observed clinical feature (Basmak, Sahin, Yildirim, Papakostas, & Kanellopoulos, 2007; Yeo et al., 2017).

Angle kappa has been measured traditionally in the clinic using a synoptophore (Basmak, Sahin, Yildirim, Papakostas, & Kanellopoulos, 2007; von Noorden & Campos, 2002). Synoptophore, also known as the major amblyoscope, is an instrument used to measure the angle of deviation in strabismus, fusional range and amblyopia therapy (She et al., 2021; Subharngkasen, 2003; Vesely & Synek, 2013). To measure angle kappa, a specially designed Maddox test slide may be inserted into one arm of the synoptophore. Patients are instructed to look at a fixation point, and the position of the corneal light reflex is observed. Next, they are instructed to fixate on the letters (right) or numbers (left) until the corneal light reflection is centred on the pupil. This angular shift in gaze position is measured as angle kappa (Basmak, Sahin, Yildirim, Saricicek, & Yurdakul, 2007). The advantages include lower equipment cost and ease of use. However, there are several limitations. Measurements are subjective and depend on the examiner's instructions and the patient's understanding of the task. This makes it difficult or unsuitable to use in young children or patients with reduced vision or special abilities (Stein et al., 1988; Wajuihian & Naidoo, 2010). Being a standalone instrument, automatic transfer of the output data to other surgical equipment or software may be difficult.

Recent advances in ophthalmic imaging allow for the measurement of angle kappa using low coherence reflectometers (Bao et al., 2023; Qin et al., 2022), aberrometers (Fu et al., 2019; Lee et al., 2019; Wang et al., 2020), Scheimpflug imaging systems (Domínguez‐Vicent et al., 2014; Qi et al., 2018), Purkinje meters (Harrer et al., 2017), ultrasound biomicroscopy (Yeo et al., 2017), and corneal topography systems (Basmak, Sahin, Yildirim, Papakostas, & Kanellopoulos, 2007; Choi & Kim, 2013; Domínguez‐Vicent et al., 2014; Gharaee et al., 2015; Hashemi et al., 2010; Yeo et al., 2017). Although these technologies provide objective measurement of angle kappa, they differ in optical principles and methods employed. For example, Orbscan II (Bausch & Lomb, Rochester, NY, USA) calculates angle kappa by measuring the radial distance between the centre of the entrance pupil and the centre of the Placido ring reflection on the cornea (Basmak, Sahin, Yildirim, Papakostas, & Kanellopoulos, 2007), Pentacam (Oculus, Wetzlar, Germany) measures the distance between corneal vertex and pupil centre (Qin et al., 2022), Lenstar (Haag‐Streit, Koniz, Switzerland) uses the X and Y coordinates of the pupil barycentre (Park et al., 2012; Qin et al., 2022), and iTrace aberrometer (Tracey Technologies Corp., Houston, TX, USA) measures the radial distance between the intercept of the visual axis and pupillary axis at the corneal plane (Qin et al., 2022). The key advantage of these measurement modalities includes fast and objective measurements of angle kappa and ocular biometry with good repeatability (Qin et al., 2022). Most of these instruments may allow data transfer and integration to a commercially available software suite for customised cataract and refractive surgery that facilitates their use in the operating room as well as in the clinic. Limitations of these instruments include the relatively higher cost of equipment and software upgrades and difficulties obtaining measurements under challenging conditions, such as in dry eyes, cloudy ocular media and eyes with tear film instability. Overall, there are significant advantages to using optical biometers and imaging devices, as evidenced by their ubiquitous usage in ophthalmology clinical practices and surgical centres.

Although degrees are appropriate units to represent angles, most of these biometers and imaging systems measure and report angle kappa as radial distance in millimetres (chord kappa). This radial distance in the Cartesian coordinate system is a close approximation of the angle measure in the polar coordinate system; therefore, angle kappa measurements can be converted from millimetres (Cartesian) to angle (polar) units or vice versa using the conversion of 1 mm = 12.5° (Bonaque‐González et al., 2021; Irsch, 2015; Riddell et al., 1994). The normal range of angle kappa values in the population is 4.51° ± 0.11° to 6.14° ± 1.44°, which corresponds to 0.24 ± 0.12 to 0.45 ± 0.20 mm (Table 1) (Basmak, Sahin, Yildirim, Saricicek, & Yurdakul, 2007; Cervantes‐Coste et al., 2022; Choi & Kim, 2013; Domínguez‐Vicent et al., 2014; Erdem et al., 2008; Fu et al., 2019; Gharaee et al., 2015; Hashemi et al., 2010; Reinstein et al., 2021; Yeo et al., 2017; Zarei‐Ghanavati et al., 2014).

**TABLE 1:** Summary of studies measuring angle kappa.

Author	Eyes, *n*	Instrument	Methods for measuring angle kappa	Angle kappa, mean ± SD	Findings
Basmak, Sahin, Yildirim, Saricicek, and Yurdakul (2007)	300	Synoptophore	Maddox test slide series A White Binding No. 16 was inserted into one arm of Synoptophore and the position of corneal light reflex was noted. Study participant fixated on letters to the left or right until the light reflex centered on the pupil This angular shift in gaze position was measured as angle kappa.	Myopic group, OD/OS: 1.74 ± 0.13°/1.91 ± 0.14° Emmetropic group, OD/OS: 2.78 ± 0.12°/3.32 ± 0.13° Hyperopic group, OD/OS: 3.44 ± 0.14°/3.84 ± 0.17°	Values obtained with OII were significantly greater vs. Synoptophore (*p* < 0.0001)
		Orbscan II corneal topography system	Angle kappa was measured as the distance between the centre of the pupil and the centre of the Placido ring reflection on the cornea.	Myopic group, OD/OS: 4.51 ± 0.11°/4.73 ± 0.11° Emmetropic group, OD/OS: 5.55 ± 0.13°/5.62 ± 0.10° Hyperopic group, OD/OS: 5.65 ± 0.10°/5.73 ± 0.10°	
Cervantes‐Coste et al. (2022)	56	OPD‐Scan III wavefront aberrometer	The location of the visual axis was estimated using topographic and wavefront measurements to find the longest light ray. Angular distance from the alignment light to the pupil centre was measured as angle kappa. (SpecialEyes, 2021)	Photopic: 0.35 ± 0.18 mm Mesopic: 0.40 ± 0.18 mm	
Choi and Kim (2013)	584	Orbscan II		Males: 4.70 ± 2.70° Females: 4.89 ± 2.14°	Angle kappa decreased with axial length and increased with age and spherical equivalent
Domínguez‐Vicent et al. (2014)	80	Orbscan II		0.43 ± 0.13 mm	OII yields significantly higher angle kappa values vs. Galilei G4 (*p* = 0.001)
		Galilei G4 topography device	Device combined a Placido disc ring to evaluate the anterior corneal surface with a rotational scan of dual‐Scheimpflug slit. Angle kappa was measured as the distance between pupil centre and the centre of the reflection of the 4 Purkinje dots.	0.27 ± 0.15 mm	
Erdem et al. (2008)	94	OPD Scan Pupillometer		Myopic group: 0.29 ± 0.14 mm Hyperopic group: 0.45 ± 0.20 mm	
Fu et al. (2019)	57	iTrace aberrometer	An iris image was captured through an infrared camera to display the centre of the pupil, the centre of the visual axis, and the centre of the limbus. Angle kappa was measured as the radial distance between the centre of the pupil and the visual axis, estimated by the centre of the first Purkinje reflex.	0.25 ± 0.12 mm	Angle kappa was significantly positively correlated with the OSI (*p* = 0.005) and significantly negatively correlated with MTF cut‐off (*p* = 0.002), Strehl ratio (*p* = 0.003), and simulated visual acuity at 100% and 20% contrast
Gharaee et al. (2015)	977	Orbscan II		All: 4.96 ± 1.38° Males: 5.00 ± 1.36° Females: 4.97 ± 1.30°	
Hashemi et al. (2010)	800	Orbscan II		5.46 ± 1.33°	Linear regression indicated a 0.015° decrease in angle kappa per year of age
Harrer et al. (2017)	395	Modified Purkinje meter	Angle kappa was measured as the angle that overlapped the centre of the pupil and the corneal reflex.	5.2 ± 2.6°	The correlation between angle kappa and age was reported (*R* ^2^ = 0.01433); increase in angle kappa was 0.03° per year.
Neuman et al. (2025)	8119	IOLMaster 700	Defined the corneal centre and pupillary centre, which were assumed to represent the optical axis and pupillary axis, respectively. Visual axis was derived from the main corneal light reflex on that capture. Angle kappa was measured as the Cartesian coordinates of the visual axis (corneal vertex) from the pupil centre.	Angle kappa offset: 0.35 ± 0.20 mm	
Reinstein et al. (2021)	750	Orbscan II		Myopic group: 5.28 ± 1.49° Emmetropic group: 5.77 ± 1.29° Hyperopic group: 6.14 ± 1.44°	
Yeo et al. (2017)	42	Orbscan II		3.98 ± 1.12°	Significant difference in angle kappa measured by the 2 methods (*p* < 0.001)
		UBM and corneal topography	Pupil centre and cornea vertex were determined using Humphrey Atlas 995 corneal topography; angle kappa was calculated based on the tangent of the angle between pupillary axis and line of sight.	3.19 ± 1.15°	
Zarei‐Ghanavati et al. (2014)	96	Orbscan II		4.97 ± 1.24°	

Abbreviations: IOL, intraocular lens; MTF, modulation transfer function; OD, right eye; OII, Orbscan II corneal topography system; OS, left eye; OSI, objective scatter index; UBM, ultrasound biomicroscopy.

Angle kappa has also been described by other names, such as angle lambda and chord mu, in the literature (Chang & Waring, 2014; Le Grand & El Hage, 1980; Montrimas et al., 2024; Nanavaty et al., 2025; Tabernero et al., 2006). This inconsistency in terminology causes confusion (Fernandez et al., 2025) and precludes meaningful comparisons of data from clinical trials. Angle lambda is the angle between the pupillary axis and line of sight. Line of sight connects the fixation point to the fovea through the centre of entrance pupil. Therefore, both line of sight and visual axis have the same reference points in the object and image space but differ on the intersection point in the path (entrance pupil centre and nodal points, respectively) (Atchison & Smith, 2000). On the other hand, chord mu is not an angle but a two‐dimensional Cartesian distance between the line of sight and the subject‐fixated, coaxially sighted, corneal light reflex axis. The magnitude of chord mu roughly correlates with the Cartesian representation (mm) of angle kappa, causing ambiguity in usage (Chang & Waring, 2014). More recently, publications refer to Chang‐Waring (CW) chord, defined as the distance between the Purkinje image reflex (first Purkinje image; PI) and the centre of the pupil, which can be measured using any ophthalmic biometer or topographer (Langenbucher et al., 2022a). Further studies will need to address the possibility of translating CW chord to angle kappa using optical models (Langenbucher et al. 2022b).

Inconsistencies in reporting angle kappa (degrees versus mm) and the use of various measurement methods can make comparison of angle kappa across studies and patient populations difficult. A study comparing angle kappa measurements from Orbscan II and a synoptophore in the same group of patients reported a strong correlation between devices (*r* = 0.932); however, significantly smaller values were reported with a synoptophore versus Orbscan II (average difference, 2.55°; Table 1) (Zhang et al., 2024). Furthermore, mean angle kappa has been reported to be consistent when Pentacam, iTrace or IOLMaster 700 were used for measuring (the differences in measurements were not statistically significant; *p* > 0.5 value for all) (Zhang et al., 2024).

Prior studies (Table 2) have reported the association of angle kappa with ocular biometry, age, sex, refractive error, ambient lighting and intraocular surgery (Atchison & Smith, 2000; Le Grand & El Hage, 1980). In a retrospective study of 436 eyes who opted for multifocal refractive lens surgery, angle kappa was negatively correlated with axial length (*r* = −0.342; *p* < 0.001) and age (*r* = 0.218; *p* < 0.001) (Choi & Kim, 2013). This negative correlation was confirmed (*r* = −0.035; *p* < 0.001) by results from a consecutive case series in patients (*n* = 217 eyes) (Baenninger et al., 2022). A univariate analysis of cross‐sectional study data in 800 eyes from a Tehran population reported a significant association of angle kappa with spherical equivalent (*r* = 0.161; *p* < 0.001), white‐to‐white distance (WTW; *r* = 0.459; *p* = 0.004) and pupil diameter (*r* = 0.273; *p* = 0.014) (Hashemi et al., 2010). Multivariate analysis revealed a significant association between WTW distance and angle kappa (*r* = 0.403; *p* = 0.009) (Hashemi et al., 2010). A large study in Chinese patients with cataract (*N* = 15 127) showed that the magnitude of angle kappa correlated positively with pupil size and crystalline lens thickness (all *p* < 0.001), but negatively with corneal power, WTW distance and anterior chamber depth (ACD) (Meng et al., 2021). Angle kappa decreased as axial length increased up to 27.5 mm, then increased with an increase in axial length, revealing a non‐linear relationship (Meng et al., 2021). Although most studies report an association with ocular biometry, three studies that used anterior segment optical coherence tomography and the Galilei anterior segment system reported no significant relationship (Harrer et al., 2017; Qi et al., 2018; Shen et al., 2023). Angle kappa is weakly correlated with age; consequently, some studies showed a negative correlation (Hashemi et al., 2010) whereas others reported no change (Berrio et al., 2010) or a positive correlation (Choi & Kim, 2013; Meng et al., 2021). No sex‐specific relationship with angle kappa has been reported (Harrer et al., 2017; Hashemi et al., 2010; Qi et al., 2018; Wang et al., 2020).

**TABLE 2:** Summary of studies that assessed correlation between angle kappa and age or optical biometry.

Author	Study design	Eyes, n	Mean age, years	Male, %	Instrument	Pre‐or post‐operative AK	Study IOLs	Angle kappa, mean ± SD	Correlation of AK with age or biometry			
									Comparison	*r*	*R* ^2^	*p*
Choi and Kim (2013)	Retrospective	436	59	49	Orbscan II	Preoperative	–	Males: 4.70° ± 2.70° Females: 4.89° ± 2.14°	AK/Age	0.218		<0.001
									AK/Axial length	−0.342		<0.001
									AK/SE	0.197		<0.001
									AK/Interpupillary distance	−0.324		<0.001
Baenninger et al. (2022)	Retrospective, consecutive case series	217	56	53	iTrace aberrometer	Preoperative	AT LISA 839MP	Positive contact lens test, 0.36 ± 0.16 mm; negative contact lens test, 0.38 ± 0.16 mm	AK/Axial length	−0.035 (−0.051, −0.019)		<0.001
Hashemi et al. (2010)	Survey (Tehran Eye Study)	800	41	39	Orbscan II	Preoperative	–	5.46° ± 1.33°	AK/Age			
									Univariable	−0.189	0.036	0.001
									AK/SE			
									Univariable	0.24	0.057	<0.001
									Multiple	0.36	0.130	<0.001
									AK/CCT			
									Univariable	0.03	0.001	0.883
									AK/ACD			
									Univariable	0.03	0.001	0.567
									AK/WTW			
									Univariable	0.18	0.031	0.004
									AK/Pupil diameter			
									Univariable	0.14	0.020	0.014
Harrer et al. (2017)	Prospective case series	395	74	40	Modified Purkinje meter	Postoperative (>3 months to <1 year)	Tecnis 1‐piece IOLs (ZA9003 or ZCB00), Tecnis 3‐piece IOLs, Superflex IOL, and others	5.2 ± 2.6°	AK/Age		0.01433	
Meng et al. (2021)	Cross‐sectional study	15 127	65	41	IOLMaster 700	Preoperative	–	0.30 ± 0.18 mm	AK/Age	0.09		<0.001
									AK/Lens thickness	0.18		<0.001
									AK/Pupil size	0.03		<0.001
									AK/WTW	−0.12		<0.001
									AK/ACD	−0.28		<0.001
									AK/CCT			
									Spearman	−0.01		<0.001
									AK/Axial length	−0.18		<0.001

Abbreviations: ACD, anterior chamber depth; AK, angle kappa; CCT, central corneal thickness; IOL, intraocular lens; SE, spherical equivalent; WTW, white‐to‐white.

Angle kappa can be influenced by lighting conditions. A study in 56 eyes reported that preoperative angle kappa measured using a wavefront aberrometer (OPD‐Scan III; Nidek Co., Ltd., Gamagori, Aichi, Japan) was about 14% larger in mesopic than in photopic conditions (mesopic, 0.40 mm; photopic, 0.35 mm). Postoperatively, angle kappa was about 33% larger in mesopic versus photopic lighting (mesopic, 0.32 mm; photopic, 0.24 mm) (Cervantes‐Coste et al., 2022). During mesopic conditions, in addition to an increase in pupil size (mesopic, 6.37 ± 0.89 mm; photopic, 4.06 ± 0.70 mm), the pupil centre shifted temporally (mean shift, 0.13 mm) toward the geometric centre of the cornea (intercept of optical axis) (Wang et al., 2020; Yang et al., 2002). However, the Purkinje image position (intercept of visual axis) is relatively stable. Consequently, the radial distance between the intercept of the visual axis and the pupillary axis increased, resulting in greater values of angle kappa in mesopic conditions (mean mesopic, 0.23 mm; mean photopic, 0.19 mm). Most eyes (85%) had pupil centre shifts of less than 0.25 mm and none more than 0.40 mm. The magnitude of pupil centre shift was independent of age and refractive error (Yang et al., 2002).

Multiple studies have demonstrated an association between angle kappa and refractive error; angle kappa was largest in hyperopes and smallest in myopes (Basmak, Sahin, Yildirim, Papakostas, & Kanellopoulos, 2007; Choi & Kim, 2013; Hashemi et al., 2010; Qazi et al., 2005; Reinstein et al., 2021; Saad et al., 2023; Tabernero et al., 2007; Wachler et al., 2003; Zarei‐Ghanavati et al., 2014). A retrospective consecutive case series measured and reported an increase in preoperative angle kappa with an increase in hyperopic refraction. Angle kappa showed a linear increase in the −5 D to +5 D spherical equivalent range, then a steeper increase beyond +5 D (Baenninger et al., 2022). A study of 548 eyes that received laser‐assisted in situ keratomileusis (LASIK) treatment after cataract surgery measured angle kappa using a Scheimpflug‐based imaging system. Angle kappa was significantly larger in hyperopic (mean ± SD, 0.31 ± 0.15 mm) compared with myopic eyes (0.21 ± 0.09 mm) (Saad et al., 2023). In a retrospective case series on 750 eyes presenting for refractive surgery, the entrance pupil centre was significantly more temporal in hyperopic eyes (mean, −0.34 mm) than emmetropic (mean, −0.28 mm) and myopic eyes (mean, 0.18 mm) (Reinstein et al., 2021). Eyes with small pupil offset (<0.15 mm), a proxy of angle kappa, were observed in 21% of myopic, 9.6% of emmetropic, and 3.2% of hyperopic eyes. Large pupil offset (>0.45 mm) was more prevalent in hyperopic (28%) than emmetropic (19%) and myopic (8.8%) eyes (Reinstein et al., 2021). This tendency of pupil offset in the temporal quadrant is in agreement with previous studies (Erdem et al., 2008; Hashemi et al., 2010; Mabed et al., 2014).

Several studies have reported a decrease in the magnitude of angle kappa after cataract surgery (Garzón et al., 2020; Sandoval et al., 2022; Wang et al., 2020). The magnitude of postoperative angle kappa was significantly smaller than the preoperative values (mean ± SD, 0.24 ± 0.12 vs. 0.30 ± 0.16 mm, respectively; *p* < 0.01) following the implantation of a diffractive trifocal IOL (AcrySof® IQ PanOptix® IOL; Alcon Inc., Fort Worth, TX, USA) (Sandoval et al., 2022). The mean vector change in angle kappa was 0.19 ± 0.14 mm, which is in agreement with a prior study with FineVision trifocal IOL (POD F; Beaver‐Visitec International, Inc., Waltham, MA, USA) (Garzón et al., 2020) and a multifocal IOL study (multiple IOL models) (Garzón et al., 2020; Wang et al., 2020). In addition, a significant correlation between preoperative and postoperative angle kappa (*r* = 0.492; *p* < 0.001) was reported (Wang et al., 2020). This case series in 81 eyes showed an increase in pupil centre shifts relative to the corneal geometric centre postoperatively. In other words, the pupil centre shifted nasally toward the Purkinje image centre, and this reduced the angular subtense between the visual and pupillary axes (angle kappa) (Wang et al., 2020).

This section highlighted the optical basis of angle kappa; measurement methods and their agreement; and the multifactorial association with ocular, demographic and environmental factors.

## 3 PART II. ANGLE KAPPA AND VISUAL OUTCOMES

There is a considerable interest in understanding the association of preoperative angle kappa and visual outcomes after MIOL implantation. This is important because (a) prior studies have hypothesized angle kappa as a contributing factor to postoperative visual disturbances (discussed in detail in Part IV), with larger angle kappa resulting in suboptimal postoperative visual outcomes (Karhanova et al., 2013; Park et al., 2012; Qi et al., 2018; Tchah et al., 2017) and (b) the magnitude of preoperative angle kappa may be a deciding factor for clinicians when considering MIOL implantation (Fu et al., 2019; Garzón et al., 2020; Karhanova et al., 2013; Park et al., 2012; Tchah et al., 2017). There are inconsistencies in the literature regarding what metrics constitute visual outcomes; therefore, in this review, visual outcomes include uncorrected and corrected visual acuity for distance, intermediate and near, range of visual acuity and depth of focus from defocus curve test and subjective reports of quality of vision.

A prospective single‐arm study (*N* = 56 eyes) found that angle kappa was not significantly associated with refraction, visual acuity or patient‐reported outcomes in patients who received AcrySof IQ PanOptix IOLs (Sandoval et al., 2022). Similar findings were reported with other diffractive optics IOLs (Cervantes‐Coste et al., 2022; Garzón et al., 2020; Kim, Eom, et al., 2020; Qi et al., 2018; Saad et al., 2023; Scheepers et al., 2023).

A retrospective consecutive case series (*N* = 217 eyes) used a multifocal contact lens test to simulate and assess tolerance of multifocal IOL visual acuity. The multifocal contact lens test was positive if patients achieved visual acuity ≤0.1 logMAR. Preoperative angle kappa was similar in the groups with a positive or negative multifocal contact lens test (0.36 and 0.38 mm, respectively). Furthermore, postoperative improvements in visual acuity were similar in eyes with small and large (≥ 0.5 mm) angle kappa (Baenninger et al., 2022).

A large‐scale retrospective review of 26 470 eyes that underwent bilateral cataract or refractive lens exchange with MIOLs (PanOptix or FineVision) reported that about 72% of the eyes (*n* = 19 059) had preoperative angle kappa >0.50 mm (Wallerstein et al., 2023), a magnitude that is considered high‐risk for MIOL implantation because of an increased risk for visual disturbances (Fu et al., 2019; Park et al., 2012; Qi et al., 2018). This study confirmed no association between the magnitude of preoperative angle kappa and postoperative sphere, cylinder, spherical and defocus equivalent, monocular uncorrected distance VA, and patient satisfaction score for distance, intermediate and near vision (Wallerstein et al., 2023).

Overall, there is a consensus among multiple studies (Table 3) that angle kappa did not influence visual outcomes following diffractive optics presbyopia–correcting IOL surgery.

**TABLE 3:** Summary of studies that assessed effects of angle kappa on visual outcomes.

Author	Study details	Eyes, *n*	Mean age, years	Male, %	Instrument	Pre‐or post‐operative AK	Study IOLs	Angle kappa, mean ± SD	Correlation of AK with visual outcomes			
									Visual outcomes	*r* (95% CI)	*R* ^2^	*p*
Baenninger et al. (2022)	Retrospective, consecutive case series	217	56	53	iTrace aberrometer	Preoperative	AT LISA 839MP	Positive contact lens test, 0.36 ± 0.16 mm; negative contact lens test, 0.38 ± 0.16 mm	UDVA	–	–	0.349
Cervantes‐Coste et al. (2022)	Prospective	56	67	39	OPD‐Scan III wavefront aberrometer	Preoperative	Liberty 677MY trifocal IOL	Photopic: 0.35 ± 0.18 mm Mesopic: 0.40 ± 0.18 mm	Postop			
									UDVA	−0.053 (−0.41, 0.32)	0.019	0.775
									CDVA	−0.128 (−0.47, 0.24)	0.029	0.491
									UIVA	−0.155 (−0.49, 0.22)	0.057	0.404
									UNVA	0.012 (−0.35, 0.37)	0.006	0.947
						Postoperative (1 month)		Photopic: 0.24 ± 0.11 mm Mesopic: 0.32 ± 0.12 mm	Postop			
									UDVA	−0.248 (−0.51, 0.05)	0.071	0.092
									CDVA	−0.187 (−0.46, 0.12)	0.045	0.209
									UIVA	−0.334 (−0.57, −0.04)	0.151	**0.022**
									UNVA	−0.269 (−0.52, 0.03)	0.098	0.067
Garzón et al. (2020)	Prospective	63	62	33	Pentacam HR topographer	Preoperative	FineVision POD F trifocal	0.24 ± 0.12 mm; AK ≤0.30 mm, 75% of eyes	Postop			
	Pupil offset defined as: low, ≤0.30 mm; high >0.30 mm								UDVA			0.855
									CDVA			0.569
									DCIVA			0.189
									UNVA (overall population)			0.289
Qi et al. (2018)	Prospective case series	89	59	53	IOLMaster and Galilei anterior segment analysis system	Preoperative	AT LISA 839MP trifocal	Group A: 0 to 0.2 mm (*n* = 29) Group B: >0.2 to 0.4 mm (*n* = 33) Group C: >0.4 mm (*n* = 27)	Postop			
									UDVA			0.069
									UIVA			0.064
									UNVA			0.142
Saad et al. (2023)	Retrospective; LASIK procedure after cataract surgery to correct residual ametropia	548	54	43	Scheimpflug‐based imaging system (Pentacam, Oculus)	Preoperative	PhysIOL Multifocal and FineVision multifocal (POD F toric, POD F, Micro F)	Overall: 0.28 ± 0.14 mm Hyperopic: 0.31 ± 15 mm Myopic: 0.21 ± 0.9 mm	CDVA			0.979
												0.238
												0.948
						Postoperative:		Overall: 0.22 ± 0.12 mm Hyperopic: 0.24 ± 12 mm Myopic: 0.17 ± 0.9 mm	CDVA			**0.05**
												**0.042**
												0.762
Sandoval et al. (2022)	Prospective, single arm; eyes stratified by AK categories: ≥ 0.4 mm (*n* = 16) ≥ 0.3–<0.4 mm (*n* = 14) <0.3 mm (*n* = 26)	56	68	25	OPD‐Scan III	Preoperative	AcrySof IQ PanOptix	0.30 ± 0.16 mm	Postop			0.87
									UDVA			0.43
									UIVA			**0.05**
									UVA			0.32
									DCIVA			0.18
									DCNVA			0.58
						Postoperative		0.24 ± 0.12 mm				
Kim, Eom, et al. (2020)	Retrospective	198	57	21	Scheimpflug‐based imaging system (Pentacam, Oculus)	Preoperative	Lentis Mplus multifocal	0.19 ± 0.10 mm	Postop			
										–	–	>0.05 >0.05
									UDVA			>0.05
									UNVA			>0.05
Velasco‐Barona et al. (2019)	Prospective, randomized study	43	68	NR	OPD‐Scan III analyser	Preoperative			Postop			
							AcrySof IQ PanOptix	0.337 ± 0.15 mm	UDVA	−0.127 (−0.52, −0.31)	0.016	0.573
									UIVA	−0.279 (−0.62, 0.16)	0.077	0.208
									UNVA	−0.095 (−0.49, 0.33)	0.009	0.671
							AT LISA tri 839MP	0.278 ± 0.13 mm	UDVA	−0.432 (−0.39, 0.87)	0.187	0.284
									UIVA	−0.360 (−0.84,0.46)	0.130	0.380
									UNVA	−0.452 (−0.87, 0.36)	0.206	0.258

*Note:* Bold values indicate statistical significance. Abbreviations: AK, angle kappa; CDVA, corrected distance visual acuity; DCIVA, distance corrected intermediate visual acuity; DCNVA, distance corrected near visual acuity; IOL, intraocular lens; NR, not reported; UDVA, uncorrected distance visual acuity; UIVA, uncorrected intermediate visual acuity; UNVA, uncorrected near visual acuity.

## 4 PART III. ANGLE KAPPA AND VISUAL QUALITY

The optical quality of the human eye can be described by measuring the ocular wavefront objectively using an aberrometer. The wavefront error (the difference between ocular wavefront and ideal wavefront) is fit with circular polynomials (Zernike) to extract orthogonal components (terms) that make up the lower‐order and higher‐order aberrations (HOA) (Thibos et al., 2002). Using the Zernike terms, various image quality metrics, such as modulation transfer function (MTF), MTF cut‐off and Strehl ratio, are constructed; some metrics also include weighting of the neural contrast sensitivity function (visual Strehl ratio) (Bühren & Kohnen, 2007). These image quality metrics describe the visual quality and patient satisfaction after cataract surgery. Several studies assessed visual quality in patients after cataract surgery; however, the association between angle kappa and visual quality is not clear (Table 4).

**TABLE 4:** Summary of studies that assessed effects of angle kappa on visual quality.

Author	Study details	Eyes, *n*	Mean age, years	Male, %	Instrument	Pre‐ or post‐operative AK	Study IOLs	Angle kappa, mean ± SD	Correlation of AK with visual quality			
									Comparison	*r* (95% CI)	*R* ^2^	*p*
Cervantes‐Coste et al. (2022)	Prospective	56	67	39	OPD‐Scan III wavefront aberrometer	Preoperative	Liberty 677MY trifocal	Photopic: 0.35 ± 0.18 mm Mesopic: 0.40 ± 0.18 mm	AK/HOAs			
									HOA total (RMS)	−0.133 (−0.50, 0.27)	0.044	0.517
									HOA corneal (RMS)	0.036 (−0.37. 0.42)	0.000	0.860
									HOA internal (RMS)	0.082 (−0.33, 0.47)	0.002	0.697
						Postoperative (1 month)		Photopic: 0.24 ± 0.11 mm Mesopic: 0.32 ± 0.12 mm	AK/HOAs			
									HOA total (RMS)	0.082 (−0.21, 0.37)	0.000	0.586
									HOA corneal (RMS)	0.204 (−0.09, 0.47)	0.014	0.169
									HOA internal (RMS)	0.206 (−0.09, 0.48)	0.068	0.169
Fu et al. (2019)	Prospective case series	57	63	41	iTrace aberrometer	Preoperative	Tecnis Symfony	0.25 ± 0.12 mm	AK/HOAs			
									OSI		0.321	**<0.001**
									MTF cut‐off		0.19	**0.002**
									Strehl ratio		0.176	**0.003**
									Simulated VA			
									100% contrast		0.185	**0.002**
									20% contrast		0.108	**0.022**
Harrer et al. (2017)	Prospective case series	395	74	40	Modified Purkinje meter	Postoperative (>3 months to <1 year)	Tecnis 1‐piece IOLs (ZA9003 or ZCB00), Tecnis 3‐piece IOLs, Superflex IOL, and others	5.2 ± 2.6°	AK/HOA			**0.04**
									Secondary astigmatism			
Qi et al. (2018)	Prospective case series	89	59	53	IOLMaster and Galilei anterior segment analysis system	Preoperative	AT LISA 839MP trifocal	Group A: 0–0.2 mm (*n* = 29) Group B: >0.2–0.4 mm (*n* = 33) Group C: >0.4 mm (*n* = 27)	AK/HOAs			
									Spherical aberration			0.573
									Coma			0.431
									Trefoil			0.394
									AK/Visual quality			
									MTF cut‐off			0.325
									Strehl ratio			0.785
									OSI			0.103
									AK >0.5 mm correlated with decreased visual quality			
									MTF cut‐off			**0.037**
									Strehl ratio			**0.048**
									OSI			0.987
Velasco‐Barona et al. (2019)	Prospective, randomized study	43	68	NR	OPD‐Scan III analyser	Postoperative	AcrySof IQ PanOptix	0.337 ± 0.15 mm	AK/HOAs			
									Total HOAs	0.371 (−0.05, 0.68)	0.138	0.088
									Internal aberration	0.304 (−0.13, 0.64)	0.092	0.168
							AT LISA tri 839MP	0.278 ± 0.13 mm	AK/HOAs			
									Total HOAs	0.173 (−0.27, 0.56)	0.030	0.226
									Internal aberration	0.240 (−0.21, 0.60)	0.57	0.146

Abbreviations: AK, angle kappa; HOA, higher‐order aberration; IOL, intraocular lens; MTF, modulation transfer function; NR, not reported; OSI, objective scatter index; RMS, root mean square; VA, visual acuity.

Studies that report a correlation between angle kappa and visual quality included a prospective case series (*N* = 395 eyes) in cataract patients implanted with Tecnis ZA9003, ZCB00 (Johnson & Johnson Vision, Irvine, CA, USA), Tecnis 3‐piece, Akreas Adapt (Bausch & Lomb, Inc., Rochester, NY, USA), Superflex (Rayner Intraocular Lenses Ltd., Hove, UK) or other IOLs. In this study, postoperative angle kappa significantly correlated with secondary astigmatism (fourth order) but not with other HOAs (measured at 6‐mm pupil) (Harrer et al., 2017). In another prospective case series in patients with cataracts who received Tecnis Symfony multifocal IOL (*N* = 48 eyes), preoperative angle kappa significantly correlated with postoperative visual quality metrics, including objective scattering index, MTF cut‐off and Strehl ratio (measured at 4‐mm pupil). From linear regression analysis, angle kappa was the strongest predictor of scatter (Fu et al., 2019).

A prospective single‐arm study in patients who were bilaterally implanted with Liberty 677MY (Medicontur Medical Engineering Ltd., Zsambek, Hungary) trifocal IOLs (*N* = 56 eyes) reported no significant correlation between preoperative angle kappa and HOAs before or after cataract surgery (Cervantes‐Coste et al., 2022). Similarly, in a prospective randomized study of patients with AcrySof IQ PanOptix or AT LISA tri 839MP (Carl Zeiss Meditec, Jena, Germany) IOLs (*N* = 43 eyes), there was no significant correlation between postoperative angle kappa and total HOAs or internal aberrations at 6 months after surgery. Interestingly, eyes with AT LISA tri 839MP experienced more aberrations than eyes with PanOptix, likely because of differences in the optical design of these IOLs (Velasco‐Barona et al., 2019).

In a prospective case series of eyes implanted with AT LISA 839MP multifocal IOLs (*N* = 89 eyes), no significant differences were reported for MTF cut‐off (cut‐off frequency at 1% of maximum MTF), Strehl ratio (area under the MTF of measured eye/area under MTF of diffraction‐limited eye), or objective scatter index when stratified by preoperative angle kappa. Preoperative angle kappa groups were ≤0.2 mm (group A, *n* = 29; 33%), >0.2 to ≤0.4 mm (group B, *n* = 33; 37%) and >0.4 mm (group C, *n* = 27; 30%) (Qi et al., 2018). However, a subgroup analysis with eyes stratified by low (≤0.1 mm; *n* = 10) and high (>0.5 mm; *n* = 9) angle kappa reported a significant difference between groups in mean ± SD MTF cut‐off (low angle kappa, 39.49 ± 6.38 cycles/degree; high, 30.28 ± 7.43 cycles/degree; *p* = 0.037) and mean ± SD Strehl ratio (low, 0.24 ± 0.06; high, 0.15 ± 0.05; *p* = 0.048), although the study did not provide a rationale for how the patients were selected for this subgroup analysis (Qi et al., 2018).

These conflicting data on the relationship between angle kappa and visual quality metrics may be due partly to differences in experimental setup and instrumentation used in the assessments, as well as differences in patient groups and study design. Additional studies are necessary to confirm the association of angle kappa and visual quality metrics.

## 5 PART IV. ANGLE KAPPA AND VISUAL DISTURBANCES

Unwanted light percepts, such as starbursts, halos, glare and negative dysphotopsia, are common complaints from patients after cataract surgery and multifocal IOL implantation (Calladine et al., 2012; Cillino et al., 2008; De Rojas et al., 2023; Kim, Na, et al., 2020; Moshirfar et al., 2023; Woodward et al., 2009). Typically, patients experience these visual disturbances in their central vision, especially under mesopic conditions (large pupils), such as night driving (Pusnik et al., 2022). These symptoms resolve spontaneously in most patients, possibly because of ocular healing or visual neuroadaptation after surgery; however, some patients continue to experience these disturbances a year after surgery (Pusnik et al., 2022). Therefore, there is considerable interest in understanding preoperative angle kappa as a risk factor for visual disturbances. In this section, data from studies that explored the association of angle kappa magnitude and visual disturbances (severity, frequency and bothersomeness) following MIOL implantation are presented (Table 5).

**TABLE 5:** Summary of studies that assessed effects of angle kappa on visual disturbances.

Author	Study details	Eyes, *n*	Mean age, years	Male, %	Instrument	Pre‐or post‐operative AK	Study IOLs	Angle kappa, mean ± SD	Visual disturbances	Correlation of AK with visual disturbances			
										Comparison	*r* (95% CI)	*R* ^2^	*p*
Cervantes‐Coste et al. (2022)	Prospective	56	67	39	OPD‐Scan III wavefront aberrometer	Preoperative	Liberty 677MY trifocal	Photopic: 0.35 ± 0.18 mm Mesopic: 0.40 ± 0.18 mm					
						Postoperative (1 month)		Photopic: 0.24 ± 0.11 mm Mesopic: 0.32 ± 0.12 mm	Halos, glare at night: 7%	Not assessed			
Garzón et al. (2020)	Prospective Pupil offset defined as: low, ≤0.30 mm; high >0.30 mm	63	62	33	Pentacam HR topographer	Preoperative	FineVision POD F trifocal	0.24 ± 0.12 mm; AK ≤0.30 mm, 75% of eyes					
						Postoperative (3 months)		0.20 ± 0.12 mm; AK ≤0.30 mm, 84% of eyes	Significant halos: 25% (14/16 patients reporting halos had AK ≤0.30 mm)	Not assessed			
Fu et al. (2019)	Prospective case series	57	63	41	iTrace aberrometer	Preoperative	Tecnis Symfony	0.25 ± 0.12 mm	Visual disturbance: 70.8% Glare: 16.7% Halos: 33.3% Glare and halos: 20.8%	Not assessed			
Holladay and Simpson (2017)	Simulation study; ray tracing analysis				Optical modelling					As AK decreases, the maximum axial distance and area for negative dysphotopsia decreases			
Sandoval et al. (2022)	Prospective, single‐arm study	56	68	25	OPD‐Scan III	Preoperative	AcrySof IQ PanOptix trifocal (toric or nontoric)	0.29 ± 0.17 mm		AK/frequency of starbursts			**0.01**
	Eyes stratified by AK categories: ≥0.4 mm (*n* = 16) 0.3–<0.4 mm (*n* = 14) <0.3 mm (*n* = 26)									AK/severity or degree of bother for glare, halos or starbursts			NS
						Postoperative		0.24 ± 0.12 mm					
Qi et al. (2018)	Prospective case series	89	59	53	IOLMaster and Galilei anterior segment analysis system	Preoperative	AT LISA 839MP trifocal	Group A: 0 to 0.2 mm (*n* = 29) Group B: >0.2 to 0.4 mm (*n* = 33) Group C: >0.4 mm (*n* = 27)	Halos: Group A, 13.8%; Group B, 24.2%; Group C, 51.8%	As AK increases, the incidence of glare and halos increases			
										Halos: Group A vs. Group B vs. Group C			<**0.05**
										Glare: Group A vs. Group C			<**0.05**
Prakash et al. (2011)	Prospective	43	59	54	Orbscan anterior segment analysis system	Preoperative	ReZoom multifocal	4.9° ± 1.5°	Halos: 27.9% Glare: 20.9%	AK/perceived severity of halos		0.26	**0.03**
										AK/glare		0.26	**0.03**
Tchah et al. (2017)	Retrospective case series	34	63	47	OPD scan aberrometer	Preoperative	Lentis Mplus multifocal	Photopic: 0.25 ± 0.13 mm	Severe glare, halos: 56%	AK/glare, halo, starburst	0.388		**0.041**
								Mesopic: 0.23 ± 0.12 mm		AK/glare, halo, starburst (bivariate logistic regression), OR = 2.16			

*Note:* Bold values indicate statistical significance. Abbreviations: AK, angle kappa; IOL, intraocular lens; NS, statistically nonsignificant; OR, odds ratio.

A number of clinical studies reported a correlation between angle kappa and visual disturbances (Holladay & Simpson, 2017; Prakash et al., 2011; Tchah et al., 2017). In a retrospective case series of patients who received unilateral segmented multifocal Lentis Mplus IOLs (Oculentis GmbH, Berlin, Germany; *n* = 34 eyes), 56% of patients reported severe glare, halos and starbursts at 6 months after implantation. These symptomatic patients had corrected distance visual acuity of 20/20. A number of factors, including preoperative photopic angle kappa (odds ratio [OR], 2.16 [95% CI, 1.07, 4.36]) and preoperative pupil centre shift (OR, 2.07 [95% CI, 0.92, 4.70]), were significantly associated with visual disturbances based on bivariate logistic regression analysis (Tchah et al., 2017).

In a prospective study of 43 eyes implanted with ReZoom multifocal IOLs (Advanced Medical Optics, Santa Clara, CA, USA), halos and glare were reported in 28% and 21% of eyes, respectively. Angle kappa was weakly correlated with glare (*R* ^2^ = 0.26; *p* = 0.03) and perceived severity of halos (*R* ^2^ = 0.26; *p* = 0.03) based on regression analysis (Prakash et al., 2011). In another prospective study of 89 eyes implanted with AT LISA 839MP, 29% of eyes had ≥1 visual disturbance at 3 months after cataract surgery (Qi et al., 2018). The incidence of halos and glare significantly correlated with an increase in angle kappa (*p* < 0.05). Furthermore, the highest incidence of photic symptoms was reported in the group with angle kappa >0.4 mm (*n* = 27/89; 30%), and the lowest incidence was reported in the group with angle kappa ≤0.2 mm (*n* = 29/89; 33%), supporting the association between angle kappa magnitude and photic phenomena (Qi et al., 2018).

These observations from clinical studies were confirmed by optical modelling studies that used ray tracing in phakic and pseudophakic eye models and reported that angle kappa, small pupils and higher IOL power were risk factors for negative dysphotopsia (Holladay & Simpson, 2017). Another modelling study evaluated a pseudophakic eye model with Tecnis and AcrySof ReSTOR multifocal IOLs and reported that angle kappa may be associated with photic phenomena, especially in eyes with shallow ACD. Specifically, the effective lens position could influence the occurrence of photic phenomena in eyes with larger angle kappa (Karhanová et al., 2015).

However, other studies found no significant correlation between larger angle kappa and visual disturbances. In a prospective study of 56 eyes implanted with AcrySof PanOptix trifocal IOL, no statistically significant differences were reported for severity or degree of bother for glare, halos, or starbursts when stratified by angle kappa magnitude (<0.3; 0.3 to <0.4; ≥0.4 mm) (Sandoval et al., 2022).

Although most patients reported visual disturbances immediately after surgery, these disturbances may not affect daily activities (Fu et al., 2019; Garzón et al., 2020). In a prospective, nonrandomised uncontrolled study of 24 patients with Tecnis Symfony IOLs, 71% reported visual disturbances (17% glare, 33% halos, and 21% both), although the correlation between angle kappa and visual disturbances was not assessed (Fu et al., 2019). Most patients (75%) reported that these photic phenomena had no effect on their daily activities. In another prospective study of 63 eyes implanted with FineVision POD, 25% of patients reported halos; most of these patients (14/16) had angle kappa ≤0.30 mm. All patients were satisfied with distance vision despite reported visual disturbances (Garzón et al., 2020), suggesting that the magnitude of angle kappa may not be a risk factor for patient dissatisfaction. Additionally, halos and glare may resolve within a few months after surgery, possibly from neuroadaptation. Although studies suggest correlations between angle kappa and patient‐reported visual disturbances, not all data support a significant association (Baumeister et al., 2005, 2009). There may be additional relationships between subjectively measured visual quality, which may also be affected by angle kappa, and patient‐reported photic phenomena. Additional studies are needed to confirm the relationship between angle kappa and visual disturbances.

## 6 PART V. ANGLE KAPPA AND IOL ALIGNMENT

Intraocular lens tilt or decentration is possible after cataract surgery and can be influenced by multiple factors, including preoperative crystalline lens position and axial length (Bonaque‐González et al., 2021). Average IOL tilt and decentration in pseudophakic eyes were reported to be 4.86° and 0.21 mm, respectively (Chen et al., 2020). IOL misalignments could result in unwanted aberrations, affecting visual outcomes and causing visual disturbances. Multifocal IOL decentration >0.75 mm can affect image quality (Soda & Yaguchi, 2012). While real‐world studies reporting prevalence on the magnitude of IOL decentration are sparse, a prior study reported >0.5 mm of decentration in about 10% of the pseudophakic population (Ale, 2011).

In a theoretical study using a pseudophakic eye model, MIOLs were centred on the pupillary axis, and critical angle kappa (angle generated when the visual axis intersects the first ring area of the MIOL) was calculated for 4 diffractive MIOLs with standard ocular parameters (Tecnis ZMB00, AcrySof ReSTOR SV2STO, AcrySof ReSTOR SB6AD1 and AcrySof ReSTOR SN6AD3). Each IOL has a critical angle to faithfully refract rays. When implanted in eyes with large angle kappa (greater than half the diameter of a multifocal IOL central optic zone) exceeding the critical angle of the IOL, the foveacentric ray may strike the edge of the IOL, causing scatter and photic phenomena (Karhanová et al., 2015). Steeper corneal curvature and shorter eyes were positively correlated with critical angle kappa (Karhanová et al., 2015). Preoperative angle kappa may be correlated with crystalline lens tilt and decentration, and may be a predictor for IOL decentration (Li et al., 2021; Shen et al., 2023). In eyes with large angle kappa, a more posterior positioning of the IOL (large effective lens position) or decentration toward the visual axis could increase the critical angle kappa and help minimize the light grazing the edge of the multifocal IOL optic zone (Karhanová et al., 2015).

A clinical study evaluated the relationship between angle kappa, MIOL centration, and subjective perception of visual disturbances in 52 eyes implanted with AcrySof ReSTOR MIOLs. IOL decentration relative to pupil centre was reported in 12 eyes. Five patients with decentred IOLs (temporal and supratemporal to pupil) reported visual disturbances when measured with a candle flame in a dark room, but not during real‐world tasks such as night driving. Few symptomatic eyes had a large angle kappa (4°) relative to the average angle kappa of 2.78° reported in this group (Karhanova et al., 2013). In a randomized prospective trial in which 40 eyes received Vivinex XY1 (Hoya Surgical Opticals, Inc., Singapore) and 45 eyes received AcrySof IQ, IOL decentration and angle kappa were positively correlated with HOAs (Chandra et al., 2022). However, this study did not assess the correlation between angle kappa and IOL decentration. Strehl ratio and contrast sensitivity were not affected by IOL decentration.

A prospective cohort study in 143 eyes with Tecnis monofocal (ZCB00) reported that horizontal and vertical angle kappa was positively correlated with horizontal and vertical IOL decentration, suggesting that angle kappa is a risk factor for IOL decentration (Xu et al., 2022). Multivariate regression analysis revealed horizontal angle kappa as a risk factor for horizontal IOL decentration (ꞵ = 0.238, *p* = 0.024); a deeper anterior chamber and larger vertical angle kappa were significantly associated with greater vertical IOL decentration (ꞵ = 0.355, *p* = 0.001) (Xu et al., 2022).

In a computational study to assess the influence of angle kappa on optical quality, corneal aberrations were measured from 20 eyes using a Pentacam for a 7‐mm pupil diameter. A pseudophakic model eye was generated based on corneal aberration data and a theoretically computed multifocal IOL wavefront. Two methods of centring the MIOL and simulating angle kappa were evaluated. In one method, the MIOL was centred at the pupil centre and displaced along with the pupil. In the second method, the MIOL was centred at the corneal apex and only the pupil was displaced. All simulations were performed using the 5‐mm pupil diameter. When angle kappa = 0, the optimal orientation of the multifocal IOL for distance vision varied for all eyes. There was a significant difference in visual Strehl ratio for distance and near vision for optimal versus worst IOL orientations, mainly caused by variability in corneal aberrations among the eyes. IOL orientation did not affect intermediate vision. When angle kappa increased, the image quality decreased for distance and near vision in both centration methods, more so when the IOL was centred on the pupil; however, image quality for intermediate vision was independent of angle kappa. When angle kappa was taken into consideration during IOL orientation calculation, image quality was similar for all viewing distances for the 2 centration methods. This optimal IOL orientation axis varied for each angle kappa (Bonaque‐González et al., 2021). In a study that evaluated effects of angle kappa‐customised implantation of rotationally asymmetric SBL‐3 MIOL (Lenstec, Inc., Christ Church, Barbados), there were no significant differences observed for near or distance visual acuity, contrast sensitivity, modulation transfer function, Strehl ratio, or patient satisfaction between the group with angle kappa‐guided IOL placement and the control group that used horizontal IOL placement. However, moderately better intermediate visual acuity was reported in eyes with angle kappa‐customised MIOL orientation versus control MIOL placement, although this effect was not statistically significant. Furthermore, the angle kappa‐customised MIOL placement resulted in significantly less vertical coma compared with the control group (*p* = 0.002) (Liu et al., 2020).

In conclusion, IOL orientation, tilt and decentration may be affected by angle kappa and will likely contribute to visual disturbances. Currently, there is a lack of consensus on where to centre the IOL: the visual axis or the pupil centre. Some surgeons prefer to centre on the Purkinje image, which roughly corresponds to where the visual axis intersects the cornea or on cornea sighting reflex, whereas others use the pupil centre (Basmak, Sahin, Yildirim, Papakostas, & Kanellopoulos, 2007; Bonaque‐González et al., 2021; Uozato & Guyton, 1987). Additional optical modelling studies could improve our understanding of the relationship between angle kappa and IOL alignment.

## 7 PART VI CLINICAL RELEVANCE AND CONCLUSIONS

Accurate assessment of preoperative angle kappa, defined as the angle between the pupillary and the visual axes, is important for successful outcomes of refractive procedures. Furthermore, it is important to differentiate angle kappa from other metrics such as angle lambda (the angle between the corneal light reflex axis and line of sight) and chord mu (radial distance between corneal light reflex and line of sight). Large angle kappa (>5.0°) has been reported in patients with pseudostrabismus (Hashemi et al., 2010). Large angle kappa may also increase the risk of decentred ablation and cause irregular astigmatism and visual disturbances (Hashemi et al., 2010). However, in some cases, angle kappa may passively compensate for residual corneal astigmatism (Espinosa et al., 2009). Shallow ACD and large angle kappa may be risk factors for photic phenomena in eyes implanted with MIOLs (Karhanová et al., 2015). Therefore, measuring angle kappa in patients with cataracts before surgery may be useful, especially in populations with certain preoperative characteristics, such as shallow ACD.

Although there is a consensus that angle kappa has no effect on visual acuity, there were conflicting reports on the correlation between angle kappa and subjective visual quality and patient‐reported disturbances. Differences in study design, angle kappa measurement methods, patient characteristics and multifocal IOL characteristics may explain the discrepancies among different studies. Additional studies optimising methodology and developing standards for angle kappa assessments are needed. Better understanding of angle kappa and its effects on postoperative outcomes, as well as of the complexities of inter‐relationships of biometry, angle kappa magnitude, and IOL alignment, may help provide optimal evaluations to achieve optimal patient satisfaction.

Based on the results of the computational study by Bonaque‐González et al., multifocal IOL orientation axis should be calculated for each patient based on their ocular biometry and taking angle kappa into consideration. When angle kappa is not known, centering the IOL on the corneal apex is the optimal approach based on this simulation study. If the surgeon prefers to centre the IOL on the pupil, measuring angle kappa and optimizing the IOL orientation axis could yield comparable visual outcomes. Variability in corneal aberrations among eyes may cause variability in the optimal IOL orientation axis for a given angle kappa, potentially resulting in some eyes experiencing visual disturbances and others remaining asymptomatic (Bonaque‐González et al., 2021). To develop successful treatments and select optimal surgical settings, future studies will need to address how these parameters balance or counteract each other. Particularly, optical modelling studies may be useful in assessing effects of angle kappa, IOL design, and anterior segment biometry on visual outcomes in patients with cataracts. Furthermore, real‐world studies that address effects of angle kappa on IOL centration and patient satisfaction are needed to provide further insight for clinicians.

## FUNDING INFORMATION

This study was funded by Alcon Research LLC. Medical writing assistance was provided by Natalia Zhukovskaya, PhD, of ICON (Blue Bell, PA, USA) and was funded by Alcon.

## CONFLICT OF INTEREST STATEMENT

Prof. Kohnen: Consultant, research and lecturing for Alcon Vision LLC, Oculus, Schwind, Staar; consultant and lecturing for Ziemer; research and lecturing for Teleon Surgical; consulting for AbbVie, Geuder, LensGen, Santen, Stadapharm, Thieme, Zeiss Meditec; lecturing for Allergan, Bausch & Lomb, Johnson & Johnson, MedUpdate, Streamed Up. Drs. Ramasubramanian and Suryakumar are employees of Alcon Research LLC.
